# Association between physical activity and cardiovascular parameters in 7-year-old children: a Chinese cross-sectional study

**DOI:** 10.1186/s12887-023-04468-2

**Published:** 2024-08-13

**Authors:** Qianchuo Wang, Hualin Wang, Bowen Du, Yujian Wu, Zhuoyan Li, Yiwei Niu, Fengxiu Ouyang, Kai Bai, Jian Wang, Kun Sun

**Affiliations:** 1 Department of Pediatric Cardiology, Xinhua Hospital, School of Medicine, Shanghai Jiao Tong University, No.1665, Kongjiang Road, Yangpu District, Shanghai, 200092 China; 2 Shanghai Key Laboratory of Children’s Environmental Health, Xinhua Hospital, School of Medicine, Ministry of Education, Shanghai Jiao Tong University, Shanghai, China

**Keywords:** Physical activity, Cardiac adaption, Cardiovascular parameters, Birth cohort study, Sex-disparity

## Abstract

**Introduction:**

Physical activity (PA) is believed to play an important part in many aspects during childhood and adolescence, especially cardiorespiratory fitness and cardiometabolic health. However, whether different levels of PA in daily life influence the structure or function of heart in school-aged children remains unknown. We aimed to investigate the association between PA and cardiovascular parameters in 7-year-old children.

**Methods:**

Follow-up data from the Shanghai Prenatal Cohort Study and the Shanghai Birth Cohort was analyzed. Perinatal information including both maternal and offspring datum was recorded. A refined questionnaire was used to evaluate the frequency and duration of children’s PA levels. Blood pressure, echocardiography, and anthropometry assessment were conducted during the follow-up of 7-year-old children.

**Results:**

Overall, high PA level was associated with higher left ventricle posterior wall thickness in diastole (LVPWd, β coefficient: 0.36, 95% CI: 0.12, 0.61), higher left ventricle mass index (LVMI, β = 0.28, 95% CI: 0.07, 0.48), mitral E/a ratio (β = 0.47, 95% CI: 0.22, 0.71) and slower heart rate (β = -0.32, 95% CI: -0.57, -0.07), compared to low PA level. Medium PA level was associated with lower diastolic blood pressure (DBP, β = -0.18, 95% CI: -0.35, -0.01). In subgroup analysis, increased relative wall thickness (RWT) was found in high PA level boys (β = 0.36, 95% CI: 0.05, 0.67), and systolic blood pressure (SBP) showed a significant decrease in high PA level girls (β = -0.42, 95% CI: -0.78, -0.06).

**Conclusions:**

This study suggested non-athlete children having higher PA level were associated with thicker left ventricle (LV) walls and better LV diastolic function, as well as slower heart rate and DBP at the age of 7. Furthermore, disparity in the association between PA level with morphological heart patterns and blood pressure existed in different sex category.

**Supplementary Information:**

The online version contains supplementary material available at 10.1186/s12887-023-04468-2.

## What is Known

Physical activity (PA) is known to play an important role in various aspects during childhood and adolescence including cardiometabolic health. Physiologically morphological and functional heart modifications are observed in both adult and pediatric athletes.

## What is New

Non-athlete children having higher PA level were associated with thicker left ventricle (LV) walls and better LV diastolic function, as well as slower heart rate and diastolic blood pressure at the age of 7. Disparity in the association between PA level with morphological heart patterns and blood pressure existed in different sex category.

## Introduction

Physical activity (PA) is defined as any bodily movement resulted from skeletal muscles contraction which leads to energy expenditure [1], including a wide range from light intensity like walking in daily life to heavy intensity like exercise training. PA has been proven to play an important role in various aspects during childhood and adolescence, including physical fitness [2], cardiometabolic health [3–5] and cognitive outcomes [6]. On the other hand, the economic burden of chronic diseases and premature deaths produced by physical inactivity is heavy. However, physical inactivity was observed in children and adolescents these years [7], for which frequent lockdowns and online lessons due to the COVID-19 pandemic were to be blamed.

Left ventricle (LV) geometry and function are both critical to cardiovascular health, and LV morphological changes could progress under adverse stimulation before functional changes [8]. Physiologically morphological and functional heart modifications are observed in both adult and pediatric athletes [9, 10]. However, data of cardiac structure and function in non-athlete children with different physical activity is limited. As far as we know, there has been no study on the association of cardiovascular parameters and PA in school-aged children.

This study was based on the platforms of Shanghai Prenatal Cohort (SPC) and Shanghai Birth Cohort (SBC). We aimed to examine the relationship between PA with the cardiovascular parameters, including blood pressure, LV geometry and function in school-aged children, and further explore if sex-disparity existed.

## Methods

### Study population

Our study included children from the SPC and SBC. Both cohorts had parallel including and excluding standards. SPC was an ongoing perspective birth cohort that enrolled 1043 Han maternal-child pairs between 2012 and 2013 at Xinhua Hospital and International Peace Maternity and Child Hospital in Shanghai. In this cohort, women over 20 years old who planned to live in Shanghai for at least 2 years were recruited. SBC recruited volunteer couples during preconception care or in early pregnancy in six collaborating hospitals from 2013 to 2016 in Shanghai, and 3692 maternal-child pairs were included. The recruit protocol of SBC has been previously described in detail [11]. In both cohorts, trained researchers conducted face-to-face questionnaire interviews at enrollment to collect social-demographic characteristics including maternal age and pre-pregnancy body mass index (BMI). Information on newborn’s sex, gestational age, birth mode (caesarean section or vaginal delivery) and anthropometric parameters was obtained at the time of delivery. Children received examinations including anthropometric measurements, blood pressure measurement and echocardiogram at the 7-year follow-up visit from 2020 to 2022. Informed written consent was obtained from parents or caregivers of all eligible children prior to their entry into the study. The research was approved by the Ethical Committee of Xinhua Hospital affiliated to Shanghai Jiao Tong University School of Medicine (XHEC-C-2012-003 and XHEC-C-2013-001-2).

### Evaluation of PA

The level of PA was evaluated through a questionnaire completed by both children and their guardians (parents or other guardians taking care of children most of the time) by face-to-face interview. PAQ-CN was used to evaluate PA of Chinese children aged 7 to 18, translated and slightly modified from The Physical Activity Questionnaire for Older Children (PAQ-C) and Adolescents (PAQ-A) [12]. In this questionnaire, PA was evaluated and calculated as a score according to general PA performance in the school and at home, together with the frequency and duration of PA in the past week. According to the PA score, children were divided into three groups: high level group (PA score more than 3.00, representing metabolic equivalents over 1500 MET-min/week and having vigorous PA in over 3 days, or metabolic equivalents over 3000 MET-min/week and having PA in over 7 days), medium level group (PA score between 2.00 and 3.00, representing having over 20 min vigorous physical activity in over 3 days, or having over 30 min moderate physical activity or walking in over 5 days, or metabolic equivalents over 600 MET-min/week and having PA in over 5 days) or low level group (PA score below 2.00, representing PA level lower than medium level). The reliability and validity of questionnaire has been examined in previous study [13].

### Anthropometric measurements

A complete anthropometric evaluation was conducted for all children according to the standardized protocol. Height was measured barefoot twice to the nearest 0.1 cm. Weight was measured with light clothing and no shoes to the nearest 0.1 kg. The average of two repeats was collected, and the third measurement was conducted if the two readings differed by 0.1 kg or 0.1 cm. BMI was calculated as weight (kg)/height^2^ (m^2^).

### Measurement of blood pressure

Blood pressure was measured from the left arm with appropriately sized cuff according to the guideline set by the American Academy of Pediatrics in 2017 [14], using the professional automatic BP monitor OMRON HBP-1300 (Omron Healthcare, Guangzhou, CHINA). Blood pressure was measured three times with a 5 min gap between replicates, after a comfortable rest of 5 min. Measurements were done by trained coordinators in duplicate and averaged.

### Measurement of cardiovascular parameters

Transthoracic echocardiography was performed for all children in a comfortable and quiet environment by two experienced echocardiographers using standard techniques in accordance with the American Society of Echocardiography [15, 16]. Quantitative measurements were obtained offline by well-trained investigators blinded to the data of subjects. Detailed information had been described previously [17]. Sex-specific 95th percentile of LVMI was used as LV hypertrophy (LVH) cutoff points [18]. LVMI = 27.30 g/m^2.7^ in girls and LVMI = 30.91 g/m^2.7^ in boys represented the sex-specific 95th percentile in the cohort.

### Statistical analysis

Continuous variables from the three groups were compared with one-way ANOVA and presented as mean ± SD. Categorical variables were compared with Pearson’s chi-square test or Fisher’s exact test and presented as percentages. To evaluate and compare the association between PA and cardiovascular parameters, 2 separate standardized linear regression models were used after Z transformations of cardiovascular parameters, and β coefficient with 95% CIs were calculated. Basic model 1 was crude model. Model 2 was adjusted for confounding factors including age, sex and BMI. Sex was excluded from the model in analyzing sex disparity. P values for trends were calculated using the median PA score of each PA level (1.74 for low PA level, 2.46 for medium PA level and 3.25 for high PA level; for analyzing sex disparity, 1.76, 2.44 and 3.20 in girls and 1.70, 2.49 and 3.28 in boys, respectively) as a quasi-continuous variable in the model. All statistical analyses were performed using Stata 16.0 software (StataCorp LP, TX). Two-sided P value less than 0.05 was considered statistically significant.

## Results

### Basic characteristics

**Fig. 1:**
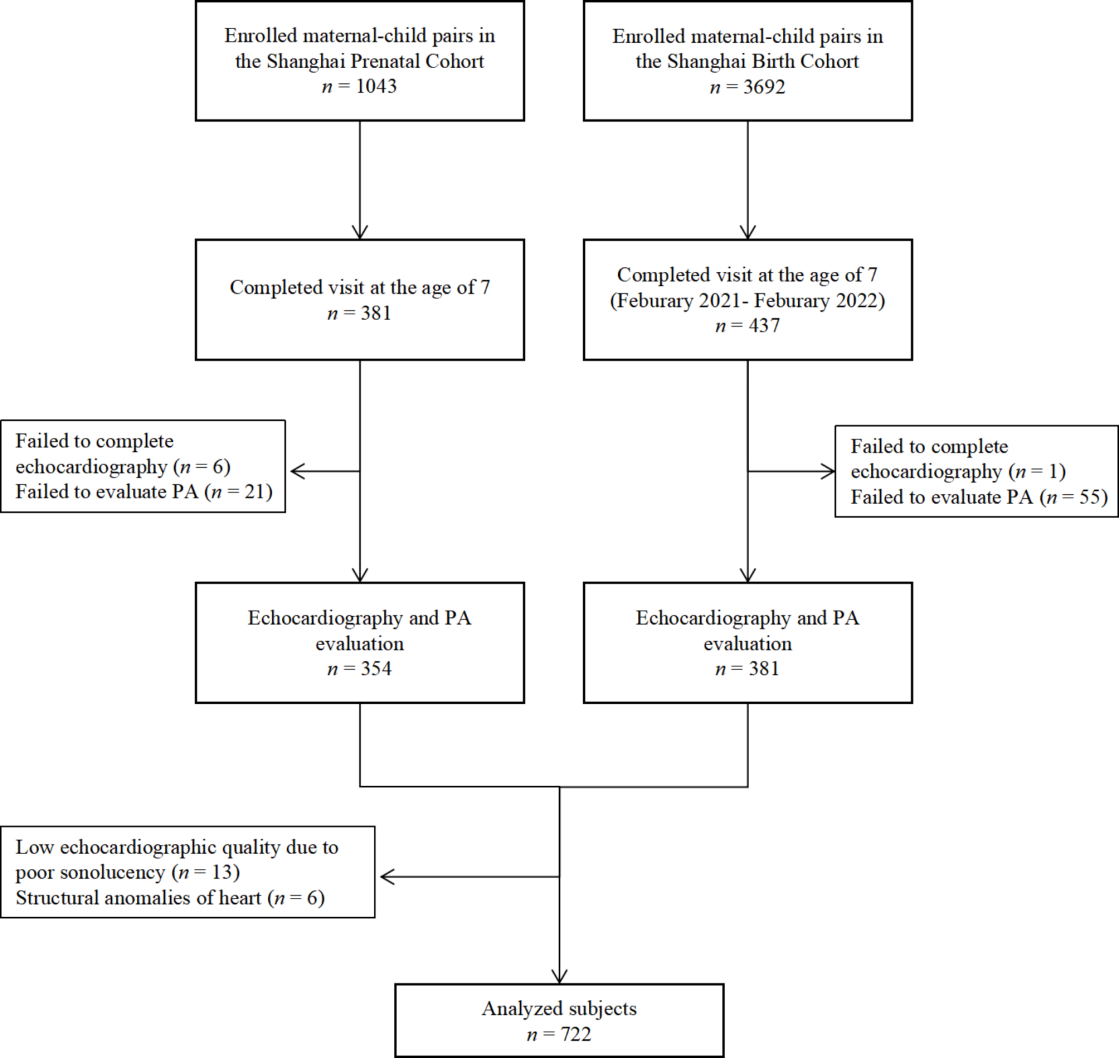
Flow diagram of participants included in this study

A total of 818 maternal-child pairs were invited in the 7-year-old visit. 96 children were excluded due to refusal to undertake echocardiogram examination or PA questionnaire survey, poor echocardiographic quality or structural anomalies of heart, and 722 children were included for data analyses at last (Fig. 1).

According to PA score, 722 children were classified into three groups: 187 children (25.9%) were at low PA level, 437 (60.5%) were at medium PA level and 98 (13.6%) were at high PA level. Maternal and child characteristics of study participants across these three groups were summarized in Table 1. The mean (± SD) PA score was 2.36 (± 0.60) for girls and 2.44 (± 0.52) for boys, reflecting more inactiveness in girls compared to boys (*P* = 0.020). No significant difference was found in BMI either at birth or at the age of 7 among these three groups.

**Table 1:** Basic characteristics of different physical activity levels

Characteristics	Physical activity level			***P***-value
	Low level N = 187	Medium level N = 437	High level N = 98	
Maternal characteristics				
Age at delivery (yr)	29.79 ± 3.82	29.85 ± 3.38	29.77 ± 3.48	0.789
Pre-pregnancy BMI (kg/m²)	21.11 ± 2.97	21.94 ± 3.70	21.39 ± 2.95	0.080
Child characteristics				
Male	88 (47%)	220 (50%)	61 (62%)	**0.045**
Age at 7-y visit (yr)	7.51 ± 0.39	7.33 ± 0.41	7.21 ± 0.40	**< 0.001**
Gestational age at delivery (wk)	38.88 ± 1.48	38.95 ± 1.80	38.93 ± 1.56	0.772
Caesarean section	137 (73%)	280 (64%)	50 (51%)	0.396
Birth height (cm)	49.79 ± 2.07	49.90 ± 1.47	49.99 ± 1.49	0.774
Birthweight (kg)	3.34 ± 0.48	3.38 ± 0.47	3.40 ± 0.54	0.678
BMI at birth (kg/m²)	13.62 ± 1.71	13.64 ± 1.33	13.81 ± 1.38	0.549
Height at 7-y visit (cm)	128.52 ± 5.87	127.15 ± 5.54	126.83 ± 6.02	**0.012**
Weight at 7-y visit (kg)	27.34 ± 6.30	26.01 ± 5.67	25.88 ± 5.74	**0.015**
BMI at 7-y visit (kg/m²)	16.41 ± 2.84	15.96 ± 2.50	15.95 ± 2.48	0.136
Physical activity score	1.71 ± 0.19	2.48 ± 0.28	3.35 ± 0.31	**< 0.001**

BMI: body mass index

Values were expressed as means ± SD or numbers (percentages). *P*-values were calculated using One-way ANOVA tests or Chi-square tests. Boldface indicates statistical significance (*p* < 0.05)

### Cardiovascular parameters in different PA level groups

Comparisons of cardiovascular parameters among three groups are demonstrated in Table 2. Thicker LV posterior wall thickness in diastole and systole (LVPWd and LVPWs) were found in children with high PA level (4.84 and 8.20 mm, respectively) than that in low PA level children (4.54 and 7.83 mm). Higher LVMI and E/a ratio were also found in high PA level children (24.09 g/m^2.7^ and 1.95) compared with low PA level children (22.25 g/m^2.7^ and 1.76). As for LV ejection fraction (LVEF), heart rate and diastolic blood pressure (DBP), decreases were shown in high PA level children (68.91%, 80.85 bpm and 60.25 mmHg) compared to low PA level children (70.31%, 84.25 bpm and 61.00 mmHg).

**Table 2:** Basic and cardiovascular characteristics of different physical activity levels at the age of 7 years

Cardiovascular parameters	Physical activity level			***P***-value
	Low level	Medium level	High level	
LV diameter				
LVPWd (mm)	4.54 ± 0.56	4.58 ± 0.80	4.84 ± 1.03	**0.006**
LVPWs (mm)	7.83 ± 1.03	7.87 ± 1.15	8.20 ± 1.20	**0.022**
LVIDd (mm)	38.71 ± 3.34	38.50 ± 3.16	38.92 ± 3.46	0.456
LVIDs (mm)	25.09 ± 2.33	24.70 ± 2.47	24.84 ± 2.64	0.211
IVSd (mm)	4.54 ± 0.46	4.54 ± 0.56	4.64 ± 0.75	0.304
IVSs (mm)	7.44 ± 1.00	7.46 ± 1.75	7.54 ± 1.10	0.858
LV geometry				
LVMI (g/m^2.7^)	22.25 ± 3.79	22.63 ± 3.97	24.09 ± 3.89	**< 0.001**
RWT (%)	23.56 ± 2.62	23.85 ± 3.94	24.72 ± 5.90	0.064
LVH, N (%)	9 (5.0%)	20 (4.6%)	7 (7.3%)	0.564
LV function				
E/a ratio	1.76 ± 0.39	1.82 ± 0.38	1.95 ± 0.41	**< 0.001**
LVEF (%)	70.31 ± 4.32	69.32 ± 4.68	68.91 ± 4.71	**0.020**
LVFS (%)	35.39 ± 3.27	35.90 ± 3.66	36.26 ± 3.43	0.115
Heart rate (bpm)	84.25 ± 10.67	81.94 ± 8.94	80.85 ± 10.78	**0.006**
Blood pressure				
SBP (mmHg)	103.74 ± 7.55	102.96 ± 7.07	103.32 ± 7.98	0.469
DBP (mmHg)	61.00 ± 5.77	59.70 ± 5.39	60.25 ± 5.77	**0.026**
Hypertension, N (%)	18 (9.6%)	34 (9.8%)	8 (8.2%)	0.746

LV: left ventricle; LVMI: LV mass index; LVPWd: LV posterior wall thickness in diastole; LVPWs: LV posterior wall thickness in systole; LVDd: LV internal diameter in diastole; LVDs: LV internal diameter in systole; IVSd: interventricular septum thickness in diastole; IVSs: interventricular septum thickness in systole; RWT: relative wall thickness; LVH: left ventricle hypertrophy; E: mitral early wave velocities; a: mitral late wave velocities; LVEF: LV ejection fraction; LVFS: LV fractional shortening; SBP: systolic blood pressure; DBP: diastolic blood pressure

Values were expressed as means ± SD or numbers (percentages). *P*-values were calculated using One-way ANOVA tests or Chi-square tests. Boldface indicates statistical significance (*p* < 0.05)

### Associations of different PA level with cardiovascular parameters in 7-year-olds

The association between different PA levels with cardiovascular parameters at 7-year-old were represented in Table 3. Positive associations were found between children with high PA level and LVPWd (β coefficient: 0.36, 95% CI: 0.12, 0.61), LVMI (β = 0.28, 95% CI: 0.07, 0.48), mitral E/a ratio (β = 0.47, 95% CI: 0.22, 0.71) ,while negative association was found in heart rate (β = -0.32, 95% CI: -0.57, -0.07). Medium PA level was significantly associated with lower diastolic blood pressure (DBP, β = -0.18, 95% CI: -0.35, -0.01). Trend analyses showed consistent results in LVPWd (β for trend = 0.22, 95% CI: 0.06, 0.38, P for trend = 0.008), LVMI (β for trend = 0.21, 95% CI: 0.06, 0.37, P for trend = 0.006), E/a ratio (β for trend = 0.29, 95% CI: 0.13, 0.45, P for trend < 0.001) and heart rate (β for trend = -0.23, 95% CI: -0.39, -0.06, P for trend = 0.006), while DBP not showing significant linear trend. Replacing BMI by height or weight in adjustment did not influence any result (Supplemental Tables 1 and 2).

**Table 3:** The association between physical activity and cardiovascular parameters at the age of 7 years

	Model 1				Model 2			
	Low level	Medium level	High level	***P*** for trend	Low level	Medium level	High level	***P*** for trend
LV diameter								
LVPWd	Reference	0.05 (-0.12,0.22)	**0.38 (0.13,0.62)**	**0.006**	Reference	0.06 (-0.11,0.23)	**0.36 (0.12,0.61)**	**0.008**
LVPWs	Reference	0.04 (-0.14,0.21)	**0.32 (0.08,0.57)**	**0.021**	Reference	-0.09 (-0.24,0.07)	0.08 (-0.15,0.30)	0.727
LVIDd	Reference	-0.06 (-0.24,0.11)	0.07 (-0.18,0.31)	0.777	Reference	0.04 (-0.11,0.19)	0.14 (-0.08,0.35)	0.230
LVIDs	Reference	-0.16 (-0.33,0.02)	-0.10 (-0.35,0.14)	0.264	Reference	0.03 (-0.12,0.18)	0.12 (-0.09,0.33)	0.288
IVSd	Reference	0.00 (-0.17,0.18)	0.17 (-0.08,0.42)	0.237	Reference	0.02 (-0.16,0.19)	0.15 (-0.10,0.40)	0.287
IVSs	Reference	0.02 (-0.16,0.19)	0.07 (-0.18,0.32)	0.612	Reference	0.02 (-0.15,0.19)	0.03 (-0.22,0.27)	0.802
LV geometry								
LVMI	Reference	0.10 (-0.08,0.27)	**0.46 (0.22,0.71)**	**0.001**	Reference	0.07 (-0.09,0.24)	**0.35 (0.12,0.59)**	**0.006**
RWT	Reference	0.07 (-0.10,0.25)	**0.29 (0.04,0.54)**	**0.029**	Reference	0.02 (-0.15,0.20)	0.23 (-0.02,0.48)	0.101
LV function								
E/a ratio	Reference	0.17 (0.00,0.34)	**0.48 (0.24,0.73)**	**< 0.001**	Reference	0.15 (-0.03,0.32)	**0.47 (0.22,0.71)**	**< 0.001**
LVEF	Reference	**-0.21 (-0.39,-0.04)**	**-0.30 (-0.55,-0.06)**	**0.008**	Reference	-0.03 (-0.19,0.13)	-0.03 (-0.36,0.19)	0.728
LVFS	Reference	0.14 (-0.03,0.32)	0.24 (0.00,0.49)	**0.040**	Reference	-0.02 (-0.19,0.14)	-0.04 (-0.27,0.20)	0.735
Heart rate	Reference	**-0.24 (-0.41,-0.07)**	**-0.35 (-0.59,-0.11)**	**0.002**	Reference	**-0.22 (-0.40,-0.05)**	**-0.32 (-0.57,-0.07)**	**0.006**
Blood pressure								
SBP	Reference	-0.11 (-0.28,0.06)	-0.06 (-0.30,0.19)	0.491	Reference	-0.04 (-0.21,0.12)	-0.02 (-0.25,0.22)	0.820
DBP	Reference	**-0.23 (-0.41,-0.06)**	-0.13 (-0.38,0.11)	0.114	Reference	**-0.18 (-0.35,-0.01)**	-0.06 (-0.30,0.19)	0.388

LV: left ventricle; LVMI: LV mass indexed to the height in m2.7; LVPWd: LV posterior wall thickness in diastole; LVPWs: LV posterior wall thickness in systole; LVDd: LV internal diameter in diastole; LVDs: LV internal diameter in systole; IVSd: interventricular septum thickness in diastole; IVSs: interventricular septum thickness in systole; RWT: relative wall thickness; E: mitral early wave velocities; a: mitral late wave velocities; LVEF: LV ejection fraction; LVFS: LV fractional shortening; SBP: systolic blood pressure; DBP: diastolic blood pressure. Results were presented as β coefficient with 95% CI. Boldface indicates statistical significance (*p* < 0.05)

Model 1: crude model. Model 2: adjusted for age, sex and BMI

### Sex disparity in the association of different PA level with cardiovascular parameters in 7-year-olds

Table 4 compared cardiovascular parameters and blood pressure among different PA groups by sex. Several parameters in each sex category remained similar variations and patterns as that in all children, including E/a ratio (*P* = 0.022), LVEF (*P* = 0.041) and heart rate (*P* = 0.010) in boys, and LVPWd (*P* = 0.026), LVMI (*P* = 0.008) and E/a ratio (*P* = 0.009) in girls. Additionally, both systolic blood pressure (SBP, *P* = 0.011) and DBP (*P* = 0.033) showed a decreasing trend with increasing PA level in girls.

**Table 4:** Sex-specific basic and cardiovascular characteristics of different physical activity levels at the age of 7

Cardiovascular parameters	Physical activity level			***P***-value
	Low level	Medium level	High level	
Boys (N = 369)				
LVPWd (mm)	4.59 ± 0.52	4.67 ± 0.66	4.77 ± 0.77	0.360
LVPWs (mm)	7.96 ± 0.98	8.08 ± 1.05	8.26 ± 1.08	0.107
LVIDd (mm)	39.50 ± 3.10	39.96 ± 2.62	39.31 ± 3.62	0.503
LVIDs (mm)	25.12 ± 2.47	25.82 ± 2.02	25.12 ± 2.66	0.124
IVSd (mm)	4.57 ± 0.40	4.62 ± 0.55	4.76 ± 0.83	0.299
IVSs (mm)	7.71 ± 0.93	7.68 ± 1.04	7.72 ± 1.16	0.915
LVMI (g/m2.7)	23.12 ± 3.58	23.82 ± 3.80	24.69 ± 4.32	0.050
RWT (%)	22.93 ± 1.97	23.67 ± 3.46	24.56 ± 5.63	0.163
LVH, N (%)	3 (3.5%)	10 (4.7%)	5 (8.3%)	0.411
E/a ratio	1.74 ± 0.37	1.81 ± 0.38	1.90 ± 0.37	**0.022**
LVEF (%)	70.55 ± 4.68	69.79 ± 4.54	68.65 ± 4.63	**0.041**
LVFS (%)	35.50 ± 3.02	36.59 ± 3.68	36.10 ± 3.28	0.098
Heart rate (bpm)	85.39 ± 12.10	81.07 ± 8.77	80.71 ± 11.10	**0.010**
SBP (mmHg)	104.38 ± 7.43	104.55 ± 6.62	105.65 ± 8.41	0.632
DBP (mmHg)	60.72 ± 5.82	59.61 ± 5.43	60.67 ± 5.45	0.444
Hypertension, N (%)	7 (8.0%)	16 (7.3%)	6 (9.8%)	0.766
Girls (N = 353)				
LVPWd (mm)	4.50 ± 0.59	4.49 ± 0.91	4.96 ± 1.38	**0.026**
LVPWs (mm)	7.72 ± 1.07	7.66 ± 1.21	8.10 ± 1.39	0.221
LVIDd (mm)	37.61 ± 3.53	37.50 ± 2.90	38.27 ± 3.10	0.290
LVIDs (mm)	24.44 ± 2.39	24.29 ± 2.40	24.35 ± 2.58	0.911
IVSd (mm)	4.52 ± 0.51	4.47 ± 0.55	4.43 ± 0.53	0.553
IVSs (mm)	7.20 ± 0.99	7.23 ± 2.22	7.23 ± 0.91	0.831
LVMI (g/m2.7)	21.49 ± 3.82	21.45 ± 3.79	23.28 ± 2.83	**0.008**
RWT (%)	24.11 ± 2.99	24.03 ± 4.36	25.00 ± 6.41	0.384
LVH, N (%)	6 (6.3%)	10 (4.6%)	2 (5.6%)	0.707
E/a ratio	1.77 ± 0.41	1.84 ± 0.37	2.03 ± 0.47	**0.009**
LVEF (%)	70.10 ± 3.99	68.86 ± 4.77	69.35 ± 4.89	0.095
LVFS (%)	35.30 ± 3.48	35.22 ± 3.52	36.53 ± 3.71	0.143
Heart rate (bpm)	83.22 ± 9.17	82.82 ± 9.04	81.08 ± 10.37	0.410
SBP (mmHg)	103.17 ± 7.65	101.34 ± 7.16	99.48 ± 5.43	**0.011**
DBP (mmHg)	61.24 ± 5.74	59.78 ± 5.35	59.55 ± 6.27	**0.033**
Hypertension, N (%)	11 (11.1%)	18 (8.3%)	2 (5.4%)	0.582

LV: left ventricle; LVMI: LV mass indexed to the height in m2.7; LVPWd: LV posterior wall thickness in diastole; LVPWs: LV posterior wall thickness in systole; LVDd: LV internal diameter in diastole; LVDs: LV internal diameter in systole; IVSd: interventricular septum thickness in diastole; IVSs: interventricular septum thickness in systole; RWT: relative wall thickness; LVH: left ventricle hypertrophy; E: mitral early wave velocities; a: mitral late wave velocities; LVEF: LV ejection fraction; LVFS: LV fractional shortening; SBP: systolic blood pressure; DBP: diastolic blood pressure

Values were expressed as means ± SD or numbers (percentages). *P*-values were calculated using One-way ANOVA tests or Chi-square tests. Boldface indicates statistical significance (*p* < 0.05)

Further investigation in adjusted linear model showed positive associations between high PA level boys and RWT (β = 0.28, 95% CI: 0.07, 0.48, β for trend = 0.22, 95% CI: 0.03, 0.42, P for trend = 0.025) additionally. LVPWd and LVMI was associated with high PA level girls (LVPWd β = 0.54, 95% CI: 0.10, 0.98; LVMI β = 0.38, 95% CI: 0.03, 0.73), however they did not show significant linear trend with PA (LVPWd β for trend = 0.26, 95% CI: -0.03, 0.54, P for trend = 0.078; LVMI β for trend = 0.18, 95% CI: -0.05, 0.41, P for trend = 0.122). A negative association between high PA level girls and SBP was shown after adjustment (β = -0.42, 95% CI: -0.78, -0.06), and the decreasing trend was found as well (β for trend = -0.29, 95% CI: -0.52, -0.06, P for trend = 0.013) (Table 5).

**Table 5:** Sex differences in the association between physical activity and cardiovascular parameters

	Model 1				Model 2			
	Low level	Medium level	High level	***P*** for trend	Low level	Medium level	High level	***P*** for trend
Boys								
LVPWd	Reference	0.11 (-0.10,0.32)	0.23 (-0.04,0.51)	0.092	Reference	0.18 (-0.03,0.39)	**0.30 (0.03,0.57)**	**0.028**
LVPWs	Reference	0.11 (-0.12,0.34)	0.26 (-0.04,0.57)	0.089	Reference	-0.02 (-0.24,0.19)	0.06 (-0.23,0.34)	0.735
LVIDd	Reference	-0.14 (-0.38,0.10)	-0.20 (-0.51,0.11)	0.189	Reference	0.02 (-0.21,0.24)	-0.04 (-0.33,0.25)	0.817
LVIDs	Reference	-0.29 (-0.53,-0.04)	-0.28 (-0.61,0.04)	0.055	Reference	-0.03 (-0.25,0.19)	0.01 (-0.28,0.30)	0.974
IVSd	Reference	0.09 (-0.17,0.35)	0.34 (0.00,0.68)	0.060	Reference	0.11 (-0.16,0.37)	0.34 (0.00,0.69)	0.057
IVSs	Reference	-0.02 (-0.19,0.16)	0.01 (-0.22,0.24)	0.956	Reference	0.00 (-0.17,0.18)	0.01 (-0.21,0.24)	0.913
LVMI	Reference	0.18 (-0.07,0.42)	**0.40 (0.07,0.72)**	**0.016**	Reference	0.18 (-0.07,0.42)	**0.37 (0.05,0.69)**	**0.024**
RWT	Reference	0.19 (-0.04,0.42)	**0.41 (0.11,0.71)**	**0.008**	Reference	0.14 (-0.09,0.38)	**0.36 (0.05,0.67)**	**0.025**
E/a ratio	Reference	0.17 (-0.07,0.40)	**0.40 (0.08,0.71)**	**0.014**	Reference	0.13 (-0.12,0.37)	**0.34 (0.02,0.67)**	**0.041**
LVEF	Reference	-0.17 (-0.42,0.09)	**-0.41 (-0.74,-0.08)**	**0.015**	Reference	0.08 (-0.15,0.31)	-0.08 (-0.38,0.22)	0.706
LVFS	Reference	**0.30 (0.06,0.55)**	0.17 (-0.15,0.19)	0.196	Reference	0.10 (-0.14,0.34)	-0.10 (-0.41,0.22)	0.637
Heart rate	Reference	**-0.44 (-0.70,-0.19)**	**-0.48 (-0.82,-0.14)**	**0.002**	Reference	**-0.42 (-0.69,-0.16)**	**-0.46 (-0.80,-0.11)**	**0.006**
SBP	Reference	0.02 (-0.22,0.26)	0.17 (-0.15,0.49)	0.321	Reference	0.15 (-0.09,0.39)	0.31 (-0.01,0.62)	0.055
DBP	Reference	-0.20 (-0.45,0.05)	-0.01 (-0.33,0.32)	0.752	Reference	-0.11 (-0.37,0.14)	0.09 (-0.24,0.42)	0.716
Girl								
LVPWd	Reference	-0.02 (-0.29,0.26)	**0.58 (0.14,1.01)**	0.050	Reference	-0.04 (-0.31,0.24)	**0.54 (0.10,0.98)**	0.078
LVPWs	Reference	-0.06 (-0.31,0.20)	0.33 (-0.07,0.74)	0.269	Reference	-0.16 (-0.38,0.07)	0.14 (-0.22,0.50)	0.912
LVIDd	Reference	-0.03 (-0.26,0.20)	0.20 (-0.16,0.57)	0.461	Reference	0.05 (-0.15,0.25)	**0.38 (0.05,0.70)**	0.052
LVIDs	Reference	-0.06 (-0.30,0.17)	-0.04 (-0.41,0.34)	0.732	Reference	0.07 (-0.13,0.27)	0.25 (-0.08,0.57)	0.152
IVSd	Reference	-0.09 (-0.32,0.14)	-0.15 (-0.52,0.22)	0.360	Reference	-0.07 (-0.30,0.15)	-0.12 (-0.48,0.25)	0.473
IVSs	Reference	0.02 (-0.27,0.32)	0.02 (-0.45,0.49)	0.904	Reference	0.03 (-0.27,0.32)	0.03 (-0.44,0.50)	0.856
LVMI	Reference	-0.01 (-0.24,0.22)	**0.40 (0.04,0.77)**	**0.093**	Reference	-0.03 (-0.24,0.19)	**0.38 (0.03,0.73)**	**0.122**
RWT	Reference	-0.02 (-0.28,0.24)	0.22 (-0.19,0.63)	0.445	Reference	-0.07 (-0.33,0.19)	0.12 (-0.30,0.53)	0.810
E/a ratio	Reference	0.17 (-0.07,0.42)	**0.66 (0.27,1.05)**	**0.002**	Reference	0.16 (-0.09,0.41)	**0.64 (0.24,1.04)**	**0.003**
LVEF	Reference	**-0.27 (-0.51,-0.03)**	-0.16 (-0.54,0.22)	0.141	Reference	-0.14 (-0.36,0.08)	0.10 (-0.25,0.45)	0.972
LVFS	Reference	-0.02 (-0.26,0.22)	0.35 (-0.03,0.73)	0.187	Reference	-0.14 (-0.36,0.09)	0.12 (-0.24,0.48)	0.941
Heart rate	Reference	-0.04 (-0.27,0.18)	-0.22 (-0.58,0.14)	0.281	Reference	-0.03 (-0.26,0.20)	-0.21 (-0.57,0.16)	0.334
SBP	Reference	**-0.25 (-0.48,-0.02)**	**-0.50 (-0.87,-0.13)**	**0.004**	Reference	**-0.22 (-0.44,0.01)**	**-0.42 (-0.78,-0.06)**	**0.013**
DBP	Reference	**-0.26 (-0.50,-0.02)**	-0.30 (-0.68,0.08)	**0.043**	Reference	-0.24 (-0.48,0.00)	-0.25 (-0.63,0.14)	0.089

LVPWd: LV posterior wall thickness in diastole; LVPWs: LV posterior wall thickness in systole; LVDd: LV internal diameter in diastole; LVDs: LV internal diameter in systole; IVSd: interventricular septum thickness in diastole; IVSs: interventricular septum thickness in systole; LVMI: LV mass indexed to the height in m2.7; RWT: relative wall thickness; E: mitral early wave velocities; a: mitral late wave velocities; LVEF: LV ejection fraction; LVFS: LV fractional shortening; SBP: systolic blood pressure; DBP: diastolic blood pressure. Results were presented as β coefficient with 95% CI. Boldface indicates statistical significance (*p* < 0.05)

Model 1: crude model. Model 2: adjusted for age and BMI

## Discussion

In this study, we demonstrated a significant association between PA and increased LVPWd, LVMI, mitral E/a ratio as well as decreased heart rate and DBP in children at the age of 7. Furthermore, disparity in the association between PA level with RWT and blood pressure existed in different sex category.

Morphological echocardiographic heart patterns of athletes have been described: predominant augmentation of wall thickness, and major cavity size in chamber dimensions in the case of prevalent static or dynamic components [9]. An observational study revealed that preadolescent athletes developed greater left ventricular mass and greater left and right ventricular chamber dimensions, while left ventricular function did not differ [10]. Another study demonstrated increased left atrium diameter, interventricular septum, and left ventricle posterior wall, diastolic diameter of the left ventricle, and right ventricle outflow tract in young athletes [19]. A randomized controlled trial showed that short-termed sports intervention in common children also resulted in changes of LV wall thickness and diastolic function [20]. In 7-year-old common pupils, LVPWd is the only significantly increased one we found among LV wall thicknesses and diameters, indicating LVPWd is one of the earliest parameters reflecting the adaption of LV to PA. We hypothesized that diastolic function improvement is associated with the increase of PA, since we observed a significant association between mitral E/a ratio and PA. LV systolic function seemed to be influenced in a rather low extent even in young athletes [21], which is consistent with our results since there was no significant association between LV systolic function and PA in non-athlete boys and girls. It was reported that greater LVMI predicted elite athletes status, while endurance elite athletes had lower heart rate and strength elite athletes had greater diastolic function in Asian physically fit young adults [22, 23]. There were similar changes of LVMI, heart rate and diastolic function in physically active children, which might be the result of mixed type of physical activity in natural condition.

PA has been linked to reducing the risk of hypertension in children and adolescents in many studies [12, 24]. However, blood pressure is regulated by multiple organs involving brain, heart, vasculature and kidney [25], and the mechanisms underlying this effect of exercise training remain undisclosed. One of the possible explanations of the positive effect of PA on blood pressure was that PA decreased the activation of the sympathetic nervous system [26, 27], and decreased heart rate should be observed together with decreased blood pressure in active children based on this assumption. A controversial finding in our study showed that decreased blood pressure did not accompany with decreased heart rate in more active girls, and decreased blood pressure was also not seen in active boys with decreased heart rate, which questioned this mechanism. Arterial remodeling should be considered as a reasonable mechanism to this effect. It was well established that changes in hemodynamic forces can lead to remodeling arteries and arterioles [28], and evidence showed that endurance trained individuals displayed a reduced arterial wall thickness and an increased lumen diameter [29]. Further studies are required to examine this mechanism in young children.

We demonstrated sex disparity in the association between cardiovascular parameters and exposure in early lifespan, which is consistent with our previous study [30]. Previous study had reported significantly larger IVSd and greater LVH prevalence in male pediatric athletes than female [31]. However, contradictory results were found in our study that girls were likely to gain more LV mass by increasing PA at the age of 7. Sex hormonal differences could be the possible explanation of these different findings. Due to the difference of hypothalamic-pituitary-gonadal axis between male and female, hormonal environment begins to differ when gonadotropin-releasing hormone is secreted from the hypothalamus to facilitate pubertal onset [32]. Some studies pointed out that sex hormones play a role in the variation of cardiac structure and function [33–35]. Our studies focused on children far before puberty, thus excluded the potential confounding effect of sex hormones.

Our cross-sectional study was based on SBC, a prospective birth cohort study platform in China, containing detailed measurement and assessment of cardiovascular parameters in early childhood, which is pioneering compared to previous epidemiology studies. Furthermore, for the first time, our study revealed sex disparity in the association between PA and cardiac remodeling in early school-aged children. The limitations in our study should also be considered. Firstly, although our study was able to adjust for several key biological factors, such as age, sex and BMI, data on other factors (diet, sleep, etc.) were not available. The observed exposure-outcome associations could change if those factors were considered. Further research is warranted encompassing a broad range of socioeconomic and lifestyle factors. Next, we chose a refined questionnaire for Chinese children as the assessment to PA due to its high convenience, where there was at risk of recall bias. Though we observed stable and consistent results in our research and the reliability of questionnaire had been examined in previous study, which made our findings credible. In addition, white-coat hypertension and masked hypertension were not considered in our study. We have standardized the procedure of measuring blood pressure to minimize the measurement error.

## Conclusions

Physically active non-athlete children were associated with thicker LV walls and better LV diastolic function as well as decreased heart rate and DBP as early as the age of 7. Disparity in the association between PA level with morphological heart patterns and blood pressure existed in different sex category. Our results suggested that there was cardiac adaption by undergoing a certain amount of intensity of daily PA instead of athletic training as early as the age of 7. It remains to be elucidated whether this adaption is beneficial for cardiovascular health in further investigations.

### Electronic supplementary material

Below is the link to the electronic supplementary material.

Supplementary Material 1

## Data Availability

The datasets used and/or analysed during the current study are available from the corresponding author on reasonable request.

## Abbreviations

DBP: diastolic blood pressure
IPAQ: International Physical Activity Questionnaire
LV: left ventricle
LVEF: LV ejection fraction
LVH: LV hypertrophy
LVMI: LV mass indexed to the height in m^2.7^
LVPWd: LV posterior wall thickness in diastole
LVPWs: LV posterior wall thickness in systole
RWT: relative wall thickness
SBC: Shanghai Birth Cohort
SBP: systolic blood pressure
SPC: Shanghai Prenatal Cohort
PA: physical activity
