# Supplementary material for: Association between physical activity and cardiovascular parameters in 7-year-old children: a Chinese cross-sectional study
Source: BMC Pediatr. 2024 Aug 13;24:522. doi: 10.1186/s12887-023-04468-2 (PMC11320774; doi:10.1186/s12887-023-04468-2)
Supplement: Supplementary file 1 — Supplementary Material 1 [file 12887_2023_4468_MOESM1_ESM.docx]

Supplemental Table 1. The association between physical activity and cardiovascular parameters at the age of 7 years, BMI replaced by height.

|  | Model 1 | | | | Model 2 | | | |
| --- | --- | --- | --- | --- | --- | --- | --- | --- |
|  | Low level | Medium level | High level | P for trend | Low level | Medium level | High level | P for trend |
| LV diameter | | | | | | | | |
| LVPWd | Reference | 0.06 (-0.11,0.23) | **0.36 (0.12,0.61)** | **0.008** | Reference | 0.06 (-0.11,0.24) | **0.37 (0.12,0.61)** | **0.008** |
| LVPWs | Reference | -0.09 (-0.24,0.07) | 0.08 (-0.15,0.30) | 0.727 | Reference | -0.10 (-0.26,0.06) | 0.06 (-0.17,0.29) | 0.867 |
| LVIDd | Reference | 0.04 (-0.11,0.19) | 0.14 (-0.08,0.35) | 0.230 | Reference | 0.03 (-0.11,0.18) | 0.12 (-0.09,0.33) | 0.275 |
| LVIDs | Reference | 0.03 (-0.12,0.18) | 0.12 (-0.09,0.33) | 0.288 | Reference | 0.03 (-0.12,0.18) | 0.13 (-0.09,0.34) | 0.273 |
| IVSd | Reference | 0.02 (-0.16,0.19) | 0.15 (-0.10,0.40) | 0.287 | Reference | 0.04 (-0.14,0.21) | 0.18 (-0.07,0.43) | 0.180 |
| IVSs | Reference | 0.02 (-0.15,0.19) | 0.03 (-0.22,0.27) | 0.802 | Reference | 0.02 (-0.16,0.19) | 0.03 (-0.22,0.27) | 0.824 |
| LV geometry | | | | | | | | |
| LVMI | Reference | 0.07 (-0.09,0.24) | **0.35 (0.12,0.59)** | **0.006** | Reference | 0.04 (-0.13,0.21) | **0.33 (0.09,0.57)** | **0.016** |
| RWT | Reference | 0.02 (-0.15,0.20) | 0.23 (-0.02,0.48) | 0.101 | Reference | 0.04 (-0.14,0.21) | 0.25 (0.00,0.50) | 0.073 |
| LV function | | | | | | | | |
| E/a ratio | Reference | 0.15 (-0.03,0.32) | **0.47 (0.22,0.71)** | **<0.001** | Reference | 0.14 (-0.04,0.31) | **0.45 (0.20,0.70)** | **0.001** |
| LVEF | Reference | -0.03 (-0.19,0.13) | -0.03 (-0.36,0.19) | 0.728 | Reference | -0.04 (-0.20,0.12) | -0.04 (-0.27,0.19) | 0.691 |
| LVFS | Reference | -0.02 (-0.19,0.14) | -0.04 (-0.27,0.20) | 0.735 | Reference | -0.03 (-0.20,0.13) | -0.06 (-0.29,0.18) | 0.628 |
| Heart rate | Reference | **-0.22 (-0.40,-0.05)** | **-0.32 (-0.57,-0.07)** | **0.006** | Reference | **-0.23 (-0.40,-0.06)** | **-0.33 (-0.58,-0.08)** | **0.005** |
| Blood pressure | | | | | | | | |
| SBP | Reference | -0.04 (-0.21,0.12) | -0.02 (-0.25,0.22) | 0.820 | Reference | -0.04 (-0.21,0.12) | -0.01 (-0.25,0.22) | 0.843 |
| DBP | Reference | **-0.18 (-0.35,-0.01)** | -0.06 (-0.30,0.19) | 0.388 | Reference | **-0.18 (-0.35,0.00)** | -0.05 (-0.30,0.20) | 0.423 |

LV, left ventricle; LVMI, LV mass indexed to the height in m2.7; LVPWd, LV posterior wall thickness in diastole; LVPWs, LV posterior wall thickness in systole; LVDd, LV internal diameter in diastole; LVDs, LV internal diameter in systole; IVSd, interventricular septum thickness in diastole; IVSs, interventricular septum thickness in systole; RWT, relative wall thickness; E, mitral early wave velocities; a, mitral late wave velocities; LVEF, LV ejection fraction; LVFS, LV fractional shortening; SBP, systolic blood pressure; DBP, diastolic blood pressure. Results were presented as β coefficient with 95% CI. Boldface indicates statistical significance (p<0.05).

Model 1: adjusted for age, sex and BMI. Model 2: adjusted for age, sex and height.

Supplemental Table 2. The association between physical activity and cardiovascular parameters at the age of 7 years, BMI replaced by weight.

|  | Model 1 | | | | Model 2 | | | |
| --- | --- | --- | --- | --- | --- | --- | --- | --- |
|  | Low level | Medium level | High level | P for trend | Low level | Medium level | High level | P for trend |
| LV diameter | | | | | | | | |
| LVPWd | Reference | 0.06 (-0.11,0.23) | **0.36 (0.12,0.61)** | **0.008** | Reference | 0.07 (-0.10,0.24) | **0.38 (0.13,0.62)** | **0.006** |
| LVPWs | Reference | -0.09 (-0.24,0.07) | 0.08 (-0.15,0.30) | 0.727 | Reference | -0.08 (-0.24,0.08) | 0.08 (-0.15,0.30) | 0.729 |
| LVIDd | Reference | 0.04 (-0.11,0.19) | 0.14 (-0.08,0.35) | 0.230 | Reference | 0.06 (-0.09,0.20) | 0.15 (-0.06,0.35) | 0.168 |
| LVIDs | Reference | 0.03 (-0.12,0.18) | 0.12 (-0.09,0.33) | 0.288 | Reference | 0.05 (-0.09,0.20) | 0.15 (-0.06,0.35) | 0.177 |
| IVSd | Reference | 0.02 (-0.16,0.19) | 0.15 (-0.10,0.40) | 0.287 | Reference | 0.04 (-0.13,0.22) | 0.19 (-0.06,0.44) | 0.156 |
| IVSs | Reference | 0.02 (-0.15,0.19) | 0.03 (-0.22,0.27) | 0.802 | Reference | 0.03 (-0.14,0.20) | 0.04 (-0.21,0.28) | 0.734 |
| LV geometry | | | | | | | | |
| LVMI | Reference | 0.07 (-0.09,0.24) | **0.35 (0.12,0.59)** | **0.006** | Reference | 0.07 (-0.10,0.24) | **0.35 (0.11,0.59)** | **0.008** |
| RWT | Reference | 0.02 (-0.15,0.20) | 0.23 (-0.02,0.48) | 0.101 | Reference | 0.03 (-0.14,0.21) | 0.25 (0.00,0.50) | 0.079 |
| LV function | | | | | | | | |
| E/a ratio | Reference | 0.15 (-0.03,0.32) | **0.47 (0.22,0.71)** | **<0.001** | Reference | 0.14 (-0.04,0.31) | **0.45 (0.20,0.70)** | **0.001** |
| LVEF | Reference | -0.03 (-0.19,0.13) | -0.03 (-0.36,0.19) | 0.728 | Reference | -0.04 (-0.21,0.12) | -0.04 (-0.27,0.19) | 0.675 |
| LVFS | Reference | -0.02 (-0.19,0.14) | -0.04 (-0.27,0.20) | 0.735 | Reference | -0.03 (-0.20,0.13) | -0.06 (-0.29,0.18) | 0.621 |
| Heart rate | Reference | **-0.22 (-0.40,-0.05)** | **-0.32 (-0.57,-0.07)** | **0.006** | Reference | **-0.23 (-0.40,-0.06)** | **-0.33 (-0.58,-0.08)** | **0.005** |
| Blood pressure | | | | | | | | |
| SBP | Reference | -0.04 (-0.21,0.12) | -0.02 (-0.25,0.22) | 0.820 | Reference | -0.03 (-0.19,0.14) | 0.01 (-0.23,0.24) | 0.978 |
| DBP | Reference | **-0.18 (-0.35,-0.01)** | -0.06 (-0.30,0.19) | 0.388 | Reference | -0.17 (-0.34,0.00) | -0.04 (-0.29,0.21) | 0.464 |

LV, left ventricle; LVMI, LV mass indexed to the height in m2.7; LVPWd, LV posterior wall thickness in diastole; LVPWs, LV posterior wall thickness in systole; LVDd, LV internal diameter in diastole; LVDs, LV internal diameter in systole; IVSd, interventricular septum thickness in diastole; IVSs, interventricular septum thickness in systole; RWT, relative wall thickness; E, mitral early wave velocities; a, mitral late wave velocities; LVEF, LV ejection fraction; LVFS, LV fractional shortening; SBP, systolic blood pressure; DBP, diastolic blood pressure. Results were presented as β coefficient with 95% CI. Boldface indicates statistical significance (p<0.05).

Model 1: adjusted for age, sex and BMI. Model 2: adjusted for age, sex and weight
